# A Case of Spinal Epidural Arteriovenous Fistula With Congestive Myelopathy: Diagnostic Value of Time-Resolved Magnetic Resonance Angiography

**DOI:** 10.7759/cureus.107421

**Published:** 2026-04-20

**Authors:** Keita Nagawa, Hirokazu Shimizu, Shinji Kakemoto, Iichiro Osawa, Eito Kozawa

**Affiliations:** 1 Radiology, Saitama Medical University Hospital, Saitama, JPN

**Keywords:** epidural venous plexus, selective catheter spinal angiography, spinal congestive myelopathy, spinal epidural arteriovenous fistula, time-resolved magnetic resonance angiography

## Abstract

Spinal epidural arteriovenous fistulas (SEAVFs) are rare types of spinal arteriovenous fistulas consisting of a connection between a distal branch of the spinal arteries and the epidural venous plexus. These fistulas cause venous engorgement, and reflux into perimedullary veins leads to spinal congestive myelopathy. The common and less invasive method to identify spinal arteriovenous fistulas is magnetic resonance angiography (MRA), and especially time-resolved MRA (TR-MRA) is desirable because it can achieve a high enough temporal resolution to image the flow dynamics of the shunt and associated vessels. Due to the rarity of the disease, the effectiveness of TR-MRA in diagnosing SEAVFs has not been well reported. In our case, a 74-year-old female patient presented with a one-week history of progressive gait disturbance and urinary incontinence. Magnetic resonance T2-weighted images of the thoracolumbar spine revealed an intramedullary hyperintensity signal area in the spinal cord with slight perimedullary flow voids. The arterial phase of TR-MRA showed an enlarged epidural venous pouch and a refluxing radicular vein supplied by branches from the right segmental arteries of T12 and L1. These findings led to the correct diagnosis of SEAVF with congestive myelopathy. The presence of arterialized epidural venous pouches is a decisive finding of SEAVFs. Our case suggests that TR-MRA can visualize these arterialized fistulas and contribute to the diagnosis of SEAVFs.

## Introduction

Spinal epidural arteriovenous fistulas (SEAVFs) are extremely rare vascular lesions that typically occur in older men over the age of 60 [1]. Although the pathophysiologic mechanism of SEAVFs remains uncertain, the possible association with neurofibromatosis, prior surgery, or trauma has been noted [1-3]. They are typically seen in the thoracic or lumbar spine. Shunts of SEAVFs are primarily fed by the dorsal somatic branch of the lumbar artery, and mainly drain into the ventral epidural venous plexus, which can cause spinal cord compression or congestive myelopathy if there is intradural reflux to perimedullary veins [3,4]. SEAVFs are treated by endovascular embolization or microsurgical resection. The choice of treatment depends on the clinical situation or the vascular anatomy of the lesions. However, it is important to distinguish SEAVFs from the more common spinal dural arteriovenous fistulas (SDAVFs) [5,6]. While both can cause myelopathy, SDAVFs occur at the dural sleeve and drain directly into intradural perimedullary veins. In contrast, SEAVFs are located within the epidural space, often involving an arterialized epidural venous pouch, and primarily drain into the epidural venous system [5,6]. The insidious nature of SEAVFs and the potential for mimicking other spinal conditions can lead to delayed diagnosis. Delayed diagnosis can result in progressive and potentially irreversible neurological deficits due to chronic venous congestion and spinal cord ischemia.

The diagnostic rate of SEAVFs has improved with advances in medical imaging technology. Digital subtraction angiography is the reference standard study for the proper diagnosis of SEAVFs [5]. Three-dimensional rotational angiography of the spinal cord provides a high-resolution view of spinal angioanatomy and allows explicit visualization of shunts and surrounding vessels of SEAVFs [6]. Computed tomography angiography (CTA) and magnetic resonance angiography (MRA) are common and less invasive methods for evaluating and characterizing vascular lesions. However, CTA and MRA cannot image the flow dynamics of shunts and associated vessels due to their low temporal resolution. Time-resolved MRA (TR-MRA) is expected to be a desirable technique that can effectively visualize the arterial supply, the nidus, and the venous drainage pattern of SEAVFs [7] because it achieves a temporal resolution high enough to separate arteries and veins in real time. While digital subtraction angiography remains the gold standard, TR-MRA provides valuable non-invasive information regarding the arterial and venous components of the fistula, allowing for improved pre-angiographic planning.

In this study, we report a case in which SEAVF was visualized by TR-MRA, which led to the correct diagnosis. We describe imaging features, including TR-MRA, that are important for the diagnosis of SEAVFs. This report highlights the diagnostic value of TR-MRA in identifying arterialized epidural venous pouches and localizing the fistula level.

## Case presentation

History

A 74-year-old female patient presented with a history of progressively worsening neurological symptoms over approximately one week. Initially, approximately 10 days prior to presentation, she experienced bilateral buttock pain and toe numbness, but remained ambulatory. Over the next few days, she developed progressive lower extremity weakness but was still able to ambulate with assistance. Approximately two days before presentation, she became unable to walk and sought medical attention from an outside physician, where lumbar spinal canal stenosis was suspected. Imaging revealed only mild spinal canal stenosis. By the time she presented to our care, she had a complete loss of ambulation, urinary incontinence, and constipation, which had gradually worsened over the preceding week. Her past medical history included diffuse large B-cell lymphoma, diagnosed seven years before, for which she had obtained complete remission after chemoradiotherapy. The rapid progression of her symptoms, particularly the development of paralysis within a week, despite the limited spinal stenosis on imaging, prompted consideration of a non-compressive etiology, such as a vascular lesion.

Neurological examination findings

Neurological examination revealed normal motor strength in the upper limbs (manual muscle testing 5/5). In the lower limbs, the iliopsoas was 3/5, quadriceps 2/5, tibialis anterior 4/5, extensor hallucis longus 4/5, flexor hallucis longus 4/5, and gastrocnemius 4/5. Deep tendon reflexes were present in the biceps, brachioradialis, and triceps (+/+) but absent in the Achilles and patellar tendons (-/-). Pathologic reflexes (Hoffmann, Tromner, Babinski) were absent. Sensory examination using a cold stimulus (0-10 scale with 10 representing normal cold sensation) demonstrated reduced sensation in bilateral lower legs, thighs, and the perianal region; sensation elsewhere (upper limbs and trunk) was normal. Anal sphincter contraction was reduced, with preserved perianal sensation.

Radiological findings

**Figure 1:**
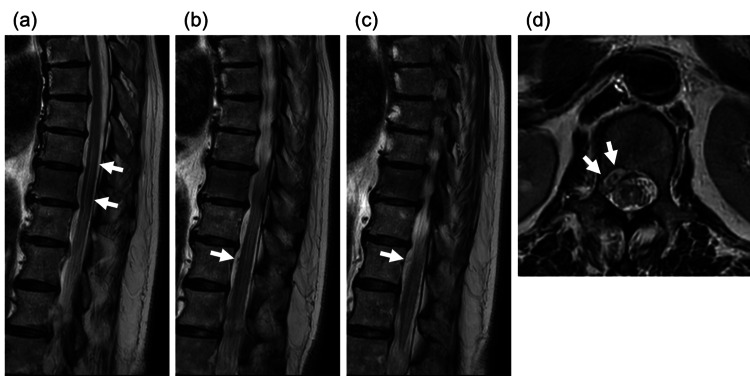
Sagittal T2-weighted images of the thoracolumbar spine (a-c) revealed long-segment intramedullary T2 hyperintensity extending from T12 to conus (a, arrows), indicating cord edema from venous congestion. Note the serpiginous perimedullary flow voids along the dorsal surface of the cord (b-c, arrows), which are suggestive of dilated perimedullary veins and should raise suspicion for a spinal arteriovenous fistula. Axial T2-weighted image (d) demonstrated an enlarged epidural venous plexus ventral to the spinal cord from T12 to L3 (arrows), a key finding in spinal epidural arteriovenous fistula that reflects the abnormal venous drainage pattern.

Sagittal T2-weighted images of the thoracolumbar spine revealed long-segment intramedullary T2 hyperintensity extending from T12 to conus, consistent with venous congestive myelopathy, with serpiginous perimedullary flow voids along the dorsal surface of the cord, suggestive of dilated venous channels (Figures 1a-1c), which has raised suspicion of spinal arteriovenous fistula. Axial T2-weighted images demonstrated an enlarged epidural venous plexus ventral to the spinal cord from T12 to L3 (Figure 1d).

**Figure 2:**
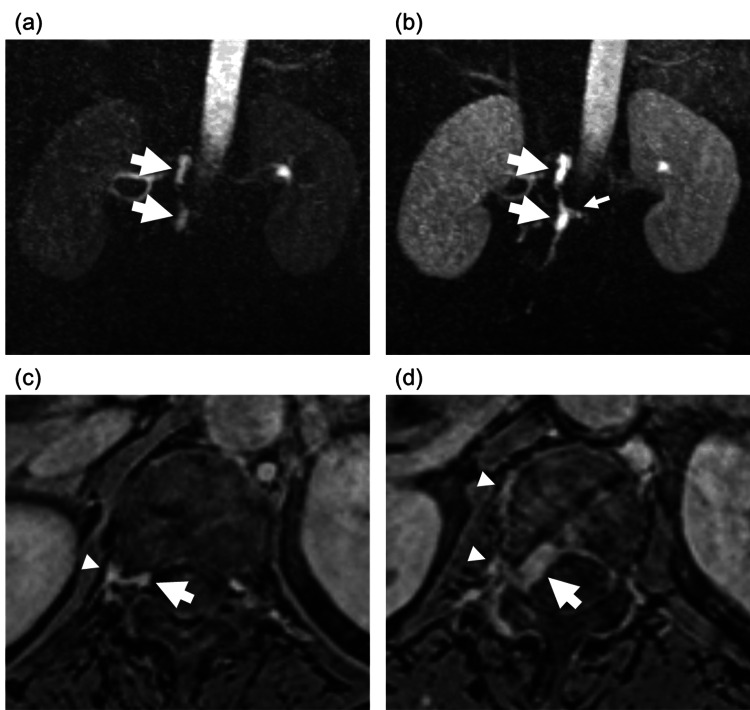
Contrast-enhanced time-resolved magnetic resonance angiography (a (30 seconds) and b (40 seconds)) revealed a right-sided epidural venous pouch on T12 and L1 (arrows), the hallmark of spinal epidural arteriovenous fistula. Axial images of the delayed contrast phase (c (T12 level) and d (L1 level)) showed the venous pouch (arrows) fed by the segmental arteries on the same levels (arrowheads). Observe the drainage into the right lateral epidural venous plexus and paravertebral venous plexus, with suggested reflux into the radiculomedullary vein (b, small arrow), demonstrating the abnormal venous drainage pattern.

In the lumbar spine, there was scoliosis and L5 spondylolisthesis without lumbar canal stenosis. Contrast-enhanced TR-MRA (TWIST (temporal resolution ~1-2 seconds); Siemens Healthcare, Erlangen, Germany) in the arterial phase revealed the right-sided epidural venous pouch on T12 and L1 (Figures 2a-2b) fed by the segmental arteries on the same levels (Figures 2c-2d). This venous pouch drained into the right lateral epidural venous plexus and paravertebral venous plexus, and the reflux into the radiculomedullary vein was also suggested (Figure 2b). These findings, particularly the presence of an enlarged epidural venous pouch and the evidence of arterial feeders originating from the epidural space, were consistent with SEAVF with intradural reflux causing congestive myelopathy, rather than SDAVF, which typically lacks an epidural venous pouch. CTA was also performed and showed similar findings to TR-MRA with no other vascular abnormalities.

Spinal angiography and endovascular treatment

**Figure 3:**
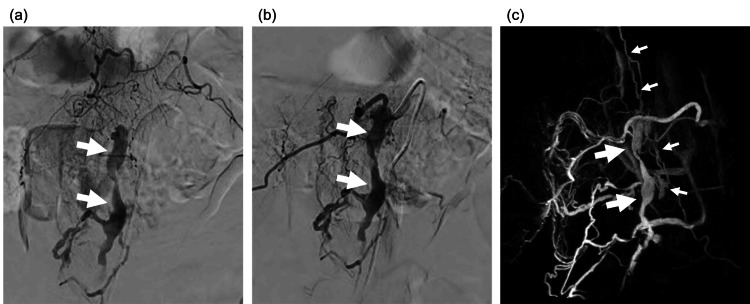
Spinal digital subtraction angiography, the right T12 (a) and L1 (b) injection, showed a spinal extradural arteriovenous fistula with the extradural venous pouch (arrows), which is the target for embolization. Volume rendered image of the CT angiography (c) confirms that the venous pouch (arrows) is connected to the radiculomedullary and perimedullary veins (small arrows), demonstrating the abnormal venous drainage pattern that causes congestive myelopathy.

Selective catheter spinal angiography showed SEAVF with the perimedullary drainage. The fistula was supplied by dorsal somatic branches from the right segmental arteries of T12 and L1, and they drained into radiculomedullary and perimedullary veins via the right-sided epidural venous pouch (Figures 3a-3c).

**Figure 4:**
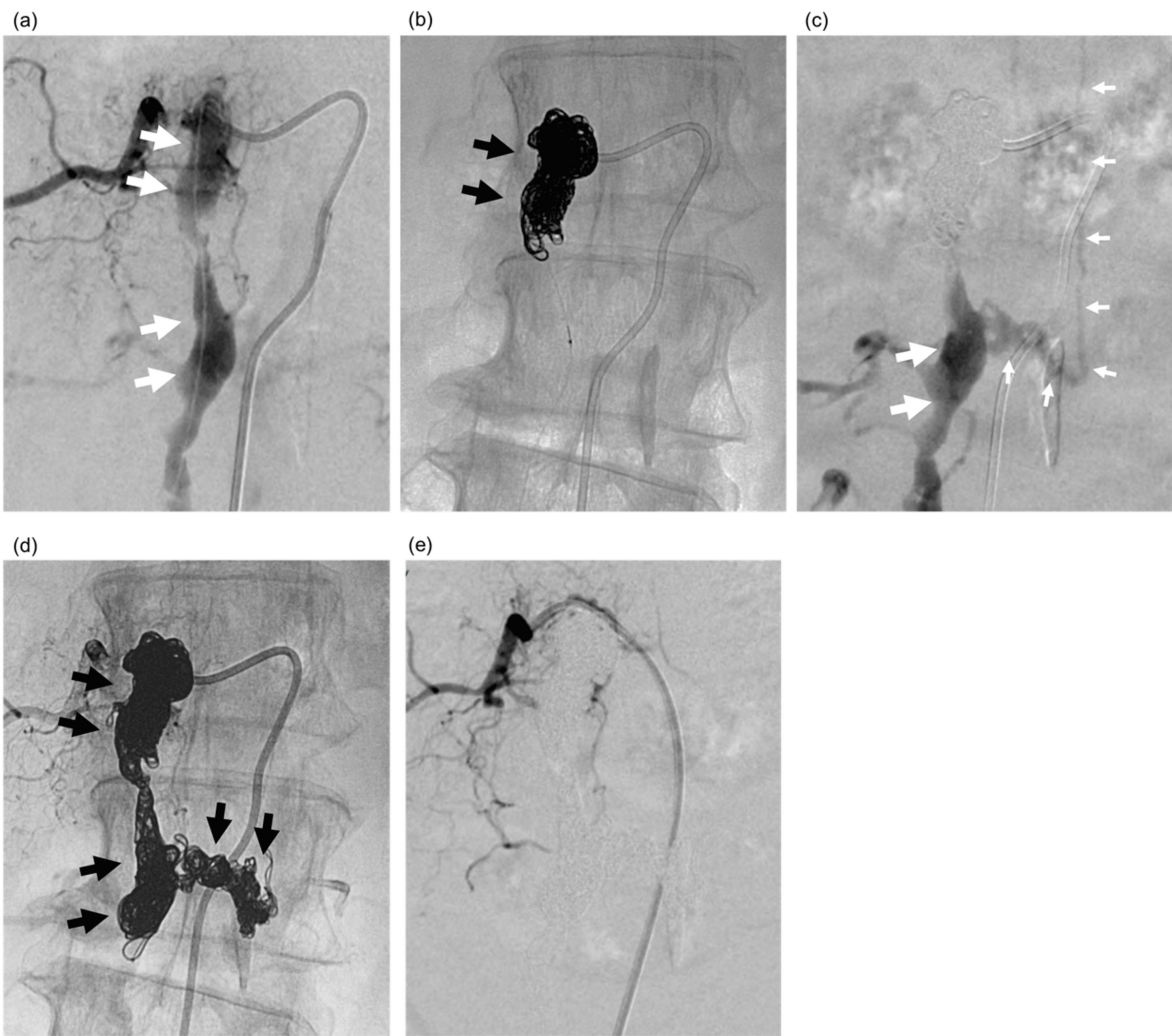
Endovascular embolization was performed. A guiding catheter was inserted into the right ascending lumbar vein from the right femoral vein via the right common iliac vein, and a Headway Duo microcatheter (Terumo Corporation, Tokyo, Japan) was advanced into the epidural venous pouch (a, white arrows). The cranial portion of the epidural venous pouch was first embolized with coils (b, black arrows), and subsequent digital subtraction angiography revealed the drainers of radiculomedullary and perimedullary veins (c, small white arrows), demonstrating the pathways that cause cord congestion. These drainers, as well as the rest of the venous pouch, were further embolized with multiple coils (d, black arrows), and 50% n-butyl-2-cyanoacrylate was finally injected for complete embolization. Postoperative selective angiography showed complete obliteration of the fistula (e). This confirms the successful exclusion of the fistula and prevention of further venous congestion.

The patient underwent endovascular embolization under general anesthesia. A guiding catheter was inserted into the right ascending lumbar vein from the right femoral vein via the right common iliac vein, and a Headway Duo microcatheter (Terumo Corporation, Tokyo, Japan) was advanced into the shunt point (Figure 4a). The epidural venous pouch, as well as the drainers of radiculomedullary and perimedullary veins, were embolized with multiple coils (Figures 4b-4d), and 50% n-butyl-2-cyanoacrylate was finally injected for complete embolization. Postoperative selective angiography showed complete obliteration of the fistula (Figure 4e).

## Discussion

SEAVFs are rare and poorly understood vascular lesions of the spine and differ from the more common SDAVFs. Classical SDAVFs form a single fistula at the dural sleeve with a single radiculomeningeal artery as a feeder, draining directly into the intradural vein and refluxing into the perimedullary venous system. However, in SEAVFs, the fistula is located in the epidural space with one or more feeding arteries and a draining epidural venous system [8]. In our case, two main feeding arteries were found, which is not uncommon in SEAVFs. Unlike SDAVFs, the epidural plexus does not directly connect with the perimedullary plexus in SEAVFs. Therefore, the clinical course of SEAVFs tends to be slower than that of SDAVFs, and patients with SEAVFs may remain asymptomatic or present later with relatively indolent symptoms such as radiculopathy [9,10]. An antireflux mechanism at the dural sleeve associated with narrowing and zigzagging radicular veins may delay exacerbation of congestion [11]. However, once the antireflux mechanism fails and the perimedullary plexus becomes engorged, the clinical presentation mirrors that of SDAVFs, manifesting as congestive myelopathy [12]. Notably, our case had a relatively rapid clinical course with worsening symptoms, likely due to acute hemodynamic alteration such as venous thrombosis or increased shunt flow [8]. As with classical SDAVFs, the goal in the treatment of SEAVFs is to occlude fistulous communication by either surgery or endovascular embolization. Percutaneous transcatheter embolization of the epidural venous pouch has been described for SEAVFs [13]. Because of the difference in angioarchitecture of SEAVFs compared with SDAVFs, the treatment can be different and more challenging. Therefore, pretherapeutic diagnosis through the use of a non-invasive imaging technique is important for subsequent angiography and treatment planning.

To better clarify the radiological differences between SEAVFs and SDAVFs, we present a structured comparison in Table 1.

**Table 1:** Radiological comparison of spinal epidural arteriovenous fistulas (SEAVFs) and spinal dural arteriovenous fistulas (SDAVFs).

Feature	SEAVFs	SDAVFs
Location	Epidural space	Dural sleeve
Number of feeders	Typically multiple	Usually single
Venous pouch	Arterialized epidural venous pouch present	Absent
Drainage pattern	Epidural venous system, ± intradural reflux	Directly to intradural perimedullary veins
Imaging hallmarks	Enlarged epidural venous plexus, and epidural venous pouch	Perimedullary flow voids

Conventional MRI findings in SEAVFs and SDAVFs are similar due to the similar underlying pathologic mechanisms of both diseases [8,12,14]. There is typically intramedullary T2 hyperintensity with edema and mild expansion of the cord in the lower thoracic region and conus medullaris, consistent with the congestive myelopathy. T2 hypointensity is seen along the surface of the cord, possibly reflecting pial capillaries containing deoxyhemoglobin secondary to venous hypertension. Furthermore, prominent vascular flow voids are seen extramedullary in the dural sac. The presence of an arterialized epidural venous pouch, which is not seen in SDAVFs, is an important diagnostic finding in SEAVFs [14]. However, this is a subtle finding that is difficult to notice on conventional MRI [14]. In our case, an enlarged epidural venous plexus was observed on non-contrast MRI, but this was not the conspicuous finding. MRA has been reported to be effective in correctly identifying arterialized epidural venous pouches and refluxing radicular veins, which can contribute to the diagnosis of SEAVFs [14]. Unlike SDAVFs, the dilated venous pouch is in close proximity to the vertebral body in SEAVFs. Therefore, MRA can be useful in such cases because it is difficult to separate from the bony cortex on CTA.

TR-MRA is a technique that was introduced by Schoenberg et al. in 1999 [15]. By visualizing the contrast agent as it passes through arterial and venous vessels, TR-MRA can provide hemodynamic information about vascular lesions [16,17]. Recent advances in MRI systems allow the acquisition time of each contrast phase of TR-MRA to be reduced to as little as one to two seconds. This enables the imaging of arterial inflow without overlapping veins, which is beneficial in the diagnosis of spinal AVFs, where the arterial inflow into the venous vessels is rapid. The previous meta-analysis showed that TR-MRA can detect the shunt level in SDAVFs with high sensitivity and specificity [16], which facilitates the subsequent conventional angiography. Although the usefulness of TR-MRA in SEAVF was not well documented, other types of MRA, including first-pass contrast-enhanced MRA, have been reported to be reliable in detecting and localizing SEAVFs [14]. TR-MRA can provide more temporal information, but has the limitation of relatively low spatial resolution [16]. In our case, TR-MRA suggested that the arterialized epidural venous pouch drained into the paravertebral venous plexus and the intradural reflux via the radiculomedullary vein, but this was slightly unclear compared to the conventional angiography. While TR-MRA improves localization compared to conventional MRI, it remains inferior to DSA in spatial resolution. Further studies are needed to examine the usefulness of TR-MRA in the diagnosis of SEAVFs.

## Conclusions

We reported a case of SEAVF with congestive myelopathy in which TR-MRA potentially aided in the diagnosis. Conventional MRI findings of SEAVFs tend to be similar to the more common SDAVFs, but TR-MRA may be a useful adjunct in identifying arterialized epidural venous pouches and refluxing radicular veins in the diagnosis of SEAVFs and guiding further angiographic evaluation. Further studies with larger cohorts are warranted to validate these findings and establish the role of TR-MRA in the management of these rare lesions.
